# Women's Perception of Using Modern Family Planning Methods in Wete District, Pemba, Tanzania

**DOI:** 10.24248/eahrj.v7i2.725

**Published:** 2023-11-30

**Authors:** Sabra S. Suleiman, Mathew D. Ndomondo, Lilian T. Mselle

**Affiliations:** a Wete District Hospital, Pemba, Tanzania; b School of Nursing, Hubert Kairuki Memorial University, Dar es Salaam, Tanzania; c School of Nursing, Muhimbili University of Health and Allied Sciences, Dar es Salaam, Tanzania

## Abstract

**Background:**

Only 11% of women use family planning in Pemba, Tanzania. Among them, 9% use modern family planning (FP). Inadequate use of modern FP in the area may result in rapid population increase and attendant negative impact on social and economic development in the country.

**Methods:**

An exploratory descriptive study was conducted in Wete District, Pemba. Thirty-eight women aged between 20 to 49 years were conveniently recruited for the study. The saturation was achieved with 4 FGDs and thematic framework guided analysis of data.

**Results:**

Modern FP methods are available and accessible in all government healthcare facilities in Pemba. However, some women perceived that modern FP are effective and others think they are ineffective in preventing pregnancy. Male dominancy, religious beliefs, polygamy, and the economy influence the low uptake of modern FP.

**Conclusion:**

Modern FP methods are widely accessible for free in Wete Pemba; however, their low uptake is influenced by social-cultural and economic factors. Community-based education on the benefit of Modern FP will facilitate positive perceptions of using modern FP and increase its use by women in Wete Pemba.

## BACKGROUND

Back in 2012, the Family Planning Initiative (FP2020) introduced a global movement to support the right of women and girls to decide freely and for themselves whether they want to have children or not, when, and how many. The main goal of FP2020 was to reach 120 million new users of modern FP methods by 2020 (the 120×20 goal) worldwide.^1,2^ Globally, 45.7% of women of reproductive age use modern FP methods.^1^ Africa has the lowest percentage of women using modern FP methods and the highest unmet need in the world. The sub-Saharan African region has limited FP services which result in high rates of unwanted pregnancies, unplanned deliveries, unsafe abortions, and maternal mortalities.^3^ FP is critical for preventing unintended pregnancy and unsafe abortions, ultimately contributing to reducing maternal and child mortality.^4^ Furthermore, FP helps to reduce poverty and empowers women and men to choose responsibly the number and spacing of children.^5^

Although the modern FP methods are useful in improving maternal and child health, some low and middle-income countries (LMICs) including Tanzania have low utilisation due to various reasons including opposition to the use of modern FP methods by husbands, fear of side effects, health concerns, dissatisfaction with sexual sensation, economic status, cultural values, lack of access to information, lack of access to health services, misconceptions about modern FP methods and religious practices.^6,7^ Other barriers to the utilization of modern FP methods are fear of side effects, and adverse reactions are significant barriers among youth.^8^

In Tanzania, the prevalence of modern FP methods use is still low, with an overall utilization of rate of 32%.^9^ This has led to childhood pregnancies and deliveries hence high fertility rates throughout reproductive life. This contributes to population growth which is thought to increase stressors on health, education systems, availability of food and clean water, natural resources, and environment, and also interfere with economic growth and development.^10^ Although modern FP methods are easily accessible at no cost in all Government health facilities in Zanzibar, their acceptance and utilisation are minimal. In Unguja for example, only 23% of all women of reproductive age use any method of FP, and 14% use modern FP methods. In Pemba, 11.2 % of women use any method of FP with only 9.1 % using modern FP methods.^11^ The overall unmet need for FP is 22% in Tanzania mainland and 28% in Zanzibar. However, the rates of unmet need for FP ranges from region to region. In North Pemba, the unmet need for FP is higher (37%) than in any other region of Tanzania.^12^ Therefore, the aim of this study was to assess the women's perception of using modern family planning methods in Wete District, Pemba, Tanzania.

## METHODOLOGY

### Study Design

This study employed an exploratory descriptive design.^13^ This design enabled the exploration of in-depth and hidden information about the use of modern FP from women of reproductive age in Wete District, Pemba.

### Study Setting

Owing to practical and logistical issues, this research was conducted in Wete District Hospital in 2018 in Wete District only. The District is located in the northern region of Pemba in Zanzibar and is bordered by Konde to the north, Chake Chake District to the south, the Indian Ocean to the east, and Kojani Island to the west. The north region was purposively selected for the study due to its low prevalence (13%), of modern FP utilisation, despite the availability and accessibility of FP services in the District. In particular, Wete District was selected because of the availability of a District Hospital that caters for many patients and clients in the District. This made Wete District conducive to exploring perceptions of women on the use of modern FP.

### Participants and Sampling

Participants for this study were women of reproductive age between 20 and 49 years, attending the reproductive and child health (RCH) clinic in Wete District hospital. This group of participants was chosen because it is the most sexually active, and therefore expected to be using modern FP.^14^ A convenient sampling strategy was used to recruit women who participated in the study. With the assistance of the staff in charge of the RCH clinic, the researcher identified eligible women who were available at RCH. The researcher then explained the purpose of the study and briefed the women about the procedure of data collection. Thereafter, the time for focus group discussions (FGDs) was arranged with women who agreed to take part in the study and were able to communicate in Kiswahili during the FGDs. One FGD was conducted per day involving 9 to 10 participants. The theoretical saturation was achieved with four FGDs that involved 38 participants in total as recommended in the literature.^15^

### Data Collection and Analysis

Four FGDs with women of reproductive age were used to collect data. The FGDs used facilitated the production of richer and more complete data and decreased the bias that could be obtained when individual interviews are used.^16^ The number of participants in each group ranged from 9–10. Discussions were conducted by the researcher and were held within the hospital premises in a quiet room to allow audibility, proper quality of the recording and to avoid interruptions. The moderator was assisted by the hired research assistant who took notes and recorded the non-verbal cues that could not be audio-recorded during the discussions. The thematic analysis framework was used to analyze data.^17^ Audio-recorded discussions were transcribed verbatim and translated from Kiswahili to English by the researcher. The researchers independently read all transcripts several times to familiarise themselves with the data and identify emerging themes as per the objectives of the study. Through reading transcribed data and field notes relevant information about the research questions was identified. Codes were generated by identifying similar segments of text describing or having similar meanings. Generated codes were marked by different colors and discussed among the research team.^18^ Thereafter, similar codes were condensed into 10 sub-themes: experience in using modern FP, decision in use of modern FP, misconception/fear of side effects, availability of modern FP, effectiveness of modern FP method, religious beliefs, social beliefs, barrier for using modern FP for women, use of traditional methods of FP and economic factors, which were further checked for similarities and differences. Finally, 3 themes as per study objectives were consolidated from the generated sub-themes: use of modern FP methods, access to modern FP services, and, social cultural, and economic factors associated with the use of modern FP).

### Ethical Consideration

Ethical approval to conduct the study was obtained from the Muhimbili University of Health and Allied Sciences (MUHAS) Institutional Review Board (ref. no. MU/PGS/SAEC/VOL.IX). A study permit was sought from the District Medical Officer in Wete District before data collection. All women signed a consent form before their participation after they were informed about the aim of the study and procedures. Each participant was told that participation was voluntary and could withdraw from the study at any time. Throughout the study conduct, confidentiality and participants' anonymity were observed. Numbers were used instead of participants' names and data was stored in a computer that had a password. Also, discussions were audio-recorded after permission was granted by the participants.

## RESULTS

Thirty-eight women participated in four FGDs. These women were of childbearing age, and their age ranged from 20 to 49 years. Their level of education was madras (only Quran) education, primary, secondary, and higher education. Most of the participants were married and were housewives.

Five superordinate themes were identified from this study, namely perceived health benefits, family gender relations, fear of side effects, availability of the FP methods, and perceived effectiveness. Influencing factors for the use of modern FP methods pointed out are religious demands, polygamy practice, and economic factors.

### Perceived Health Benefits

#### Having a healthy family by using modern FP methods

Participants agreed that using modern FP facilitates women to have a limited number of children, spaced pregnancies, and healthy mothers and their children:

*“I have used modern FP methods for a long time … for me, I think the modern FP is good to use for the mothers because you may space children and it's easy to keep your family healthy” (Participant 9, FGD 2).*

*Family gender relations and decision to use modern FP methods*

Women reported that although they are the ones who use FP, the decision to use the method of FP is commonly made by their husbands and most of the time their husbands' decision is not to use modern FP methods.

*“Most of our husbands, discussing modern FP methods with their partners is a waste of time simply because they are against the use of modern FP methods. They claim that the use of modern FP methods causes a lot of problems for most women. The worst thing is that they want to deliver us morning and evening”* (Participant 2, FGD 4).

#### Fear of Side Effects of Modern FP Methods

The study revealed that some women of reproductive age perceived and experienced the side effects as a barrier to using modern FP methods. Reluctance to use modern FP methods was evidenced in all FGDs. Modern FP methods are not acceptable in the area because they are considered to cause adverse effects. The most common side effects which were mentioned by the respondents were weight changes, bleeding, infertility, cancer, and lack of sexual desire. Additionally, it can cause water leaking into and the swelling of the abdomen, as described by one participant:

*“Modern FP methods are not good for most women because they cause many health problems such as hypertension, diabetic, irregular menstruation or even cancer due to their complications”* (Participant 5, FGD4).

#### Availability of Modern FP Methods

The availability and knowledge of modern FP methods are often the most important consideration for the woman when choosing a method. If the method is easily available and perceived as effective women can use it. This study found that modern FP methods were available in all government health facilities in Wete District. During the discussions, women agreed that modern family planning services in the government health facilities and clinics around the District were available for free:

*“It is a good thing to see that all government hospitals and health centers have modern FP methods …)”* (Participant 3, FGD1).

*“I tell you modern FP methods are plenty and easy to access in clinics”* (Participant 4, FGD 2).

However, women complained about waiting for quite a long time before they could get services at the health facilities due to so many activities of health care providers. Some clients reported waiting three to five hours in the clinic on several occasions for them to be served and on some occasions, they had to leave without being attended to:

*I went to the clinic early Thursday but I met the clinic full of people waiting for different services, while health care providers were only two, and after waiting for a long period, at last, I decided to go back home without any service. Since then I have not used any modern FP…”* (Participant 6, FGD1).

#### Perceived Effectiveness of Modern FP Methods

Modern FP methods are known to be effective in preventing pregnancy. However, participants had different perspectives on the effectiveness of FP methods which was of great concern. Participants expressed that some women used modern FP methods but did not prevent them from getting pregnant:

*“I have seen some women who use modern FP methods conceive without being aware, and even without resuming their menstrual periods. So, some modern FP methods are not effective to prevent pregnancy”* (Participant 9, FGD 2).

### Factors Influencing the Use of Modern FP Methods

The researcher was particularly interested to explore sociocultural factors for using modern FP methods. It was learned that social-cultural and economic factors commonly affected the use of modern FP methods among women in society.

#### Religious Beliefs

Religion as part of culture brought a different perspective into the discussion. Some religions prohibit the use of modern FP methods. Therefore, other women reported that using modern FP was against their religious beliefs. Participants explained that children are gifts from God and people must receive everything God gives them and thought that taking modern FP methods is a sin.

“*Although many people in the community are increasingly using modern FP methods, they have to know that their religion does not allow them to do so. God is the only one who decides for everything from conception to death, and it is very wrong to interfere with this process with modern FP methods brought to us”* (Participant 4, FGD 3).

*“… only God has the power to prevent pregnancy alone. We can use modern FP methods but still, we end up getting pregnant”* (Participant 4, FGD1).

Furthermore, other women during the discussion thought that there is no need for women to use modern FP to reduce the number of children in the family because in their culture, having many children is prestige in the family. If a married woman has few children and is considered by her family that she is not fertile:

*“Our culture does not permit women to use modern FP methods because God knows and gives us everything … ”* (Participant 4, FGD1).

#### Polygamy System

It was learned from the discussions that the polygamous lifestyle which is more prominent in the area of study influences the use of modern FP methods among women. Women shared that polygamous wives do not commonly use modern FP methods due to jealousy and competition of having more children, especially boys. Bearing many children in a polygamous family would mean security for the mother on the husband's wealth:

*“It is quite obvious that most women in a polygamous relationship are competing with co-wives to have more children…”* (Participant 4, FGD 2).

Women further shared that those who are employed usually use modern FP methods because maternity leave is given after every three years. Therefore, to avoid pregnancy for three years the woman opts to use modern FP methods:

“*As government employees, if you don't use modern FP methods, you are likely to become pregnant within a short period. I was required to report to work after 40 days for this child because my first child is only one year and two months”* (Participant 4, FGD3).

#### Myths and Misconceptions about Modern FP Methods

Participants reported not using modern FP methods because they are not safe and that even healthcare providers do not use them and ask their relatives not to use them:

*“…. We hear people talking in the streets that health care providers tell us to use these methods but health workers themselves do not use them at all, that's why you can see them delivering frequently like rats. This is because they know these methods have problems for the users. Worse enough there are some medical doctors and other healthcare workers who tell their relatives and friends that modern FP methods are generally not good because they may cause a lot of health problems. My neighbor was told by her brother who is a medical doctor that she should never use any kind of modern FP methods because they are not safe”* (Participant 4, FGD 2).

#### Economic factors

The findings showed that the use of modern FP methods is good for women because women get enough time for caring for children and engage in income-generating activities that can improve their living conditions.

“*I started using modern FP when I saw a child of a woman who was using modern FP is in good health. Also, the mother was in good health with a good income to educate her children. These made me start using modern FP methods”* (Participant 5, FGD 1)

However, other women disagreed with this notion and believed that the improved social economic status of the family entirely depends on God's will:

*“Using modern FP is good and important, but we must accept that everything is from God, when one economic situation is hard like failing to pay rent, buy food and other things, it cannot be solved by using modern FP”* (Participant 5, FGD 3).

## DISCUSSION

This qualitative study, set out to explore the women's perception of using modern FP methods and associated factors. It contributes to the available literature on drivers of utilization of modern FP method particularly in LMICs. The results are discussed according to the key identified themes to reflect the perceptions of the participating women namely, perceived health benefits, family gender relations, fear of side effects, availability of the FP methods, perceived effectiveness; while influencing factors are religious demands, polygamy, and economic factors.

### Perceived Health Benefits

The study has shown that women of reproductive age who have experience in using modern FP methods support the use of modern FP as it helps to space their children, improve their own and their babies' health, and increase their income. This is consistent with the study done in Kenya which reported that high usage of modern FP methods among women of reproductive age was mainly motivated by modern FP role in regulating family sizes and the health of mothers and their children.^19^ As a result, mothers participated in development activities to increase family income.

### Family Gender Relations

The study found that gender relations play an important role in decision-making and is an essential aspect of the social context of reproductive health. Husbands dominate wives when the decision about family size is to be made; this influences the uptake of modern FP methods. This finding is similar to the study which showed that decisions and practices of modern FP are determined by the level of male support and involvement. Regardless of the knowledge and positive attitude towards the use of modern FP methods, the uptake of the methods by the majority of women in Africa is influenced by men. For example, 90% of women in Ghana reported limited access to modern FP methods services due to rejection from their husbands. Furthermore, 65.8% of married women in central Tanzania would use modern FP methods if their husbands support them.^20^ This exposes married women to several risks including emotional and physical violence if discovered by their husbands. Furthermore, when men discover that their wives are using modern FP methods e.g. implants and intrauterine contraceptive devices, they will force their wives to remove them. This means that women's power in reproductive health services particularly in the use of modern FP methods is based on gender power relations and women are submissive to their husbands. Likewise, this is consistent with a study conducted in Kenya,^21^ which found that lack of agreement on contraceptive use, reproductive intentions, and husbands' attitudes towards their role as decision-makers as barriers for married women to use the modern FP methods. Another study that is in line with this study was conducted in Malaysia where it was reported that men opposed the use of modern FP methods by their spouses because when the wives started using modern FP methods they did not consult their husbands.^6^ In addition, the findings are similar to those found in Sub-Sahara Africa whereby men have a great influence on women's decision-making and lessen women's power of taking responsibility for their health status as a right.^22^

### Fear of Side Effects

The study has found that users and non-users feared side effects of modern FP methods. Participants revealed that they experienced one or more side effects from the modern FP methods they were using. These include irregular menstrual periods and increased bleeding, weight gain, weight loss, headache, dizziness, and leaking of vaginal fluid. These problems reduce or stop the use of modern FP methods. This is consistent with what was observed in ethnic Chinese women living in the UK where their attitudes and perceptions towards modern FP methods were negative because they felt that modern FP methods affect the menstrual cycle, which is unnatural for the body and therefore undesirable.^23^ This study finding corroborates another study conducted in Kenya which found that women of reproductive age perceived that when using modern FP methods one could give birth to a child with a congenital disability.^8^

Contrary to our study findings, research conducted in Uganda demonstrated slightly positive perceptions toward the use of modern FP methods among women of reproductive age.^21^

Availability of the Modern FP Methods The study findings showed that availability of modern FP methods is adequate in every health facility and every woman of reproductive age can easily access any kind of modern FP methods free of charge. However, some women reported not using modern FP methods. This is consistent with the study conducted in rural Malawi which found that the availability of modern FP methods did not influence women of reproductive age to use them.^24^ This suggests that availability of modern FP methods alone does not determine the use of these services; hence other factors influence decision on whether or not to take up modern FP methods. The study further showed that some women stop using modern FP methods because of being ignored by healthcare providers when they seek healthcare or advice about modern FP methods related problems. Similar results were reported in the study conducted in Malaysia that health providers played an important role in using modern FP methods. It was found that healthcare providers had a negative attitude toward the use of modern FP methods among young women, hence discouraging them from using them.^30^ This attitude of some healthcare providers may weaken the efforts of the health sector toward the achievement of the FP2020 Goal, which was to reach 120 million new users of modern contraceptive methods by 2020 worldwide.

### Perceived Effectiveness

With respect to effectiveness of modern FP methods, some participants reported conceiving while using modern FP methods. This correlates with the study carried out in Kenya which indicated the effectiveness of modern FP methods to be 97% where instructions had been adhered to 3% of women, became pregnant while using modern FP methods particularly when instructions on how to use them are not followed.^25^ This has negative effect on women's health because the woman will not be physically and psychologically prepared to be pregnant and hence opt for unsafe termination of pregnancy.

Religious demands is one of the influencing factors mentioned to influence the use modern FP methods. The results showed that women of reproductive age were obliged by their religious teachings which prohibit the use of modern FP methods. Using modern FP methods is a big sin; it is doing things against God's will. Children are a gift from God. Furthermore, using modern FP methods is culturally not accepted because women of reproductive age are obliged to deliver as many children as possible. To some extent, this means that the use of modern FP methods is affected by social and cultural practices including religion. These findings are similar to those found in Afghanistan where sex and sex-related subjects are regarded as religious matters.^26^ Another study was conducted in Ethiopia to find barriers to using modern FP methods due to religious beliefs, where Ethiopian Orthodox Christians or Muslim followers reported that their religion prohibits birth control or contraceptive use.^19^ Related findings also were found in Pakistan where men do not allow their wives to use modern FP methods due to religious reasons.^27^

Polygamy practice was considered to be incompatible with modern FP methods. The study found that polygamy practice makes women compete to have many children to earn the husband's love and respect from society, hence avoiding the use of modern FP methods. In addition, participants perceive that the use of modern FP methods is a westernised way of life (for the United States of America and Europe). In their belief, planning for the number and spacing of children in the family is not African culture, and not even a Zanzibar way of life. Furthermore, the participants strongly believe that children are a gift from God, so when they are born God knows how they will earn their living. This correlates with the study conducted to find social-cultural factors influencing the use of modern FP methods. The study showed that social norms that encourage large family size, male child preference, prohibition of contraceptive use, and a tendency of women to rely on men to make all houses hold decisions negatively influence the uptake of modern FP methods.^28,29^

### Strengths and Limitations of the Study

The strength of this study was that to ensure credibility of the findings the researcher spent adequate time in the study setting to create rapport and gain participants' trust.^31^ Kiswahili, a native language was used during data collection which enhanced participation during the discussion. The framework of analysis was used during the analysis process and data accuracy was validated by member checks.^32^ Participants' quotes were included to support researchers' interpretations of the data and direct quotes enhanced credibility of the findings.^33^ The limitations are non-inclusion of men to provide a wider perspective, dependence on the honesty of respondents which is not necessarily guaranteed, recall bias whereby participants might not have been to remember fully examples of what they said. Finally, the study involved just a few respondents from one health facility, hence the results cannot be generalized over the whole country. However, it provides a foundation based upon which quantitative studies can be undertaken to get more insight into the phenomenon, including determining association various factors with use of modern FP methods.

## CONCLUSION

This study was an endeavor to investigate perceptions of women of reproductive age on modern FP methods in Wete District, Pemba. The study concludes that although modern FP methods are widely available and accessible for free in Wete District, Pemba, the uptake is greatly influenced by religious beliefs, the polygamy system, and economic factors. It is therefore recommended that community-based education on the importance and benefit of using modern FP methods be intensively provided to women of reproductive age. This would reduce myths and facilitate positive perceptions of using modern FP methods. Furthermore, another study should be conducted including participants from southern Zanzibar where the rate of use of modern FP methods is 29% to identify and better understand enablers of modern FP methods use among women of reproductive age in this region.

## References

[1] Cahill N, Sonneveldt E, Stover J, et al. Modern contraceptive use, unmet need, and demand satisfied among women of reproductive age who are married or in a union in the focus countries of the FP 2020 initiative: a systematic analysis using the Family Planning Estimation Tool. Lancet. 2018;391(10123):870–882. doi:10.1016/S0140-6736(17)33104-5 29217374 PMC5854461

[2] Starbird E, Norton M, Marcus R. Investing in family planning: Key to achieving the sustainable development goals. Glob Heal Sci Pract. 2016;4(2):191–210. doi:10.9745/GHSP-D-15-00374 PMC4982245 27353614

[3] Ouedraogo L, Habonimana D, Nkurunziza T, et al. Towards achieving the family planning targets in the African region: a rapid review of task sharing policies. Reprod Health. 2021;18(1):1–12. doi:10.1186/s12978-020-01038-y 33485339 PMC7825212

[4] Nowell LS, Norris JM, White DE, Moules NJ. Thematic Analysis: Striving to Meet the Trustworthiness Criteria. Int J Qual Methods. 2017;16(1):1–13. doi:10.1177/1609406917733847

[5] Dansou J. The benefits of family planning (FP) use in Benin: An application of the Demographic Dividend Model (DemDiv). Gates Open Res. 2019;3(1110):1–12. doi:10.12688/gatesopenres.12906.1 31723727 PMC6826173

[6] Muanda M, Ndongo PG, Taub LD, Bertrand JT. Barriers to modern contraceptive use in Kinshasa, DRC. PLoS One. 2016;11(12):1–13. doi:10.1371/journal.pone.0167560 PMC5132197 27907138

[7] Atchison CJ, Cresswell JA, Kapiga S, et al. Sexuality, fertility and family planning characteristics of married women aged 15 to 19 years in Ethiopia, Nigeria and Tanzania: A comparative analysis of cross-sectional data. Reprod Health. 2019;16(1):1–14. doi:10.1186/s12978-019-0666-0 30665470 PMC6341518

[8] Ochako R, Mbondo M, Aloo S, et al. Barriers to modern contraceptive methods uptake among young women in Kenya: a qualitative study. BMC Public Health. 2015;15(1):118. 25884675 10.1186/s12889-015-1483-1 PMC4336491

[9] Yussuf MH, Elewonibi BR, Rwabilimbo MM, Mboya IB, Mahande1 MJ. Trends and predictors of changes in modern contraceptive use among women aged 15–49 years in Tanzania from 2004–2016: Evidence from Tanzania Demographic and Health Surveys. PLoS One. 2020;15(6 June):1–14. doi:10.1371/journal.pone.0234980 PMC7323946 32598371

[10] Ndayizigiye M, Fawzi MCS, Lively CT, Ware NC. Understanding low uptake of contraceptives in resource-limited settings: a mixed-methods study in rural Burundi. BMC Health Serv Res. 2017;17(1):1–12. doi:10.1186/s12913-017-2144-0 28298207 PMC5353936

[11] TDHS. Tanzania 2015–16 Demographic Health Survey and Malaria Indicator Survey. Tanzania 2015–16 Demogr Heal Surv Malar Indic Surv. Published online 2016:24.

[12] TDHS 2015–16. Tanzania National Bureau of statistics. Tanzania demorgraphic and health indicators. Published online 2015:630.

[13] Akhtar MI. Research design. Res Soc Sci Interdiscip Perspect. 2014;(September):68–84.

[14] Humphries H, Osman F, Knight L, Abdool Karim Q. Who is sexually active? Using a multi-component sexual activity profile (MSAP) to explore, identify and describe sexually-active high-school students in rural KwaZulu-Natal, South Africa. BMC Public Health. 2019;19(1):1–13. doi:10.1186/s12889-019-6602-y 30885161 PMC6423781

[15] D. L. M. Focus Groups as Qualitative Research. Sage Publications; 1997.

[16] Then KL, Rankin JA, Ali E. Focus group research: what is it and how can it be used? Can J Cardiovasc Nurs. 2014;24(1):16–22. 24660275

[17] Javadi M, Zarea K. Understanding Thematic Analysis and its Pitfall. J Client Care. 2016;1(1):34–40. doi:10.15412/j.jcc.02010107

[18] Birt L, Scott S, Cavers D, Campbell C, Walter F. Member Checking: A Tool to Enhance Trustworthiness or Merely a Nod to Validation? Qual Health Res. 2016;26(13):1802–1811. doi:10.1177/1049732316654870 27340178

[19] Orach CG, Otim G, Aporomon JF, et al. Perceptions, attitude and use of family planning services in post conflict Gulu district, northern Uganda. Confl Health. 2015;9(1):1–11. doi:10.1186/s13031-015-0050-9 26265935 PMC4531537

[20] Msovela J, Tengia–Kessy A, Rumisha SF, Simba DO, Urassa DP, Msamanga G. Male partner approval on the use of modern contraceptive methods: factors determining usage among couples in Kibaha district, Tanzania. Contracept Reprod Med. 2020;5(1):1–7. doi:10.1186/s40834-020-00107-8 32318272 PMC7164238

[21] R. M. Kei, The Use of Modern Contraceptives Among Women of Child Bearing Age Attending MCH/FP Clinic at Uasin Gishu Sub-County Hospital, Uasin-Gishu County, Kenya. Sci J Public Heal. 2015;3(4):500. doi:10.11648/j.sjph.20150304.17

[22] Prata N, Fraser A, Huchko MJ, et al. Women's empowerment and family planning: A review of the literature. J Biosoc Sci. 2017;49(6):713–743. doi:10.1017/S0021932016000663 28069078 PMC5503800

[23] Verran A, Evans S, Lin DJ, Griffiths F. The experiences and perceptions of family planning of female Chinese asylum seekers living in the UK. J Fam Plan Reprod Heal Care. 2015;41(2):122–127. doi:10.1136/jfprhc-2013-100764 24744056

[24] Afriyie P, Tarkang EE. Factors influencing use of modern contraception among married women in Ho west district, Ghana: Descriptive cross-sectional study. Pan Afr Med J. 2019;33:1–11. doi:10.11604/pam|.2019.33.15.17500 PMC6620060 31312331

[25] Mayhew SH, Colombini M, Kimani JK, Tomlin K, Warren CE, Mutemwa R. Fertility intentions and contraceptive practices among clinic-users living with HIV in Kenya: A mixed methods study. BMC Public Health. 2017;17(1):1–15. doi:10.1186/s12889-017-4514-2 28679389 PMC5498886

[26] Osmani AK, Reyer JA, Osmani AR, Hamajima N. Factors influencing contraceptive use among women in Afghanistan: Secondary analysis of Afghanistan Health Survey 2012. Nagoya J Med Sci. 2015;77(4):551–561. doi:10.18999/nagjms.77.4.551 26663934 PMC4664587

[27] Mustafa G, Azmat SK, Hameed W, et al. Family Planning Knowledge, Attitudes, and Practices among Married Men and Women in Rural Areas of Pakistan: Findings from a Qualitative Need Assessment Study. Int J Reprod Med. 2015;2015(10):1–8. doi:10.1155/2015/190520 PMC4569791 26421316

[28] Knorr M. Promoting gender equality within family planning programs. 2016.

[29] Chanthakoumane K, Maguet C, Essink D. Married couples' dynamics, gender attitudes and contraception use in Savannakhet Province, Lao PDR. Glob Health Action. 2020;13(sup2). doi:10.1080/16549716.2020.1777713 PMC7480449 32741343

[30] Daniele MAS, Cleland J, Benova L, Ali M. Provider and lay perspectives on intra-uterine contraception: A global review. Reprod Health. 2017;14(1):1–11. doi:10.1186/s12978-017-0380-8 28950913 PMC5615438

[31] Shenton AK. Strategies for ensuring trustworthiness in qualitative research projects. Educ Inf. 2016;22(2):12–15.

[32] Creswell JW. Note de lecture : Qualitative inquiry and research design. Choosing among five approaches (3e éd.). London : Sage. Res gate. 2015;1(November):4.

[33] Noble H, Smith J. Issues of validity and reliability in qualitative research. Evid Based Nurs. 2015;18(2):34–35. doi:10.1136/eb-2015-102054 25653237

